# Nerve growth factor: what can surgeons and oncologists learn from a neurological and psychological biomarker?

**DOI:** 10.1186/s10020-025-01333-z

**Published:** 2025-08-09

**Authors:** Fei Xiong, Ben-li Xiao, Qi Wang, Kun Liu, Hong-wei Wu, Chao Jing, Kui-nan Tong, Zhong-tao Zhang, Wei Guo

**Affiliations:** 1 Department of General Surgery, Beijing Friendship Hospital, Capital Medical University, Beijing, China; 2 State Key Lab of Digestive Health, Beijing, China; 3 National Clinical Research Center for Digestive Diseases, Beijing, China; 4 Department of Gastroenterology and Hepatology, Erasmus MC-University Medical Center Rotterdam, Rotterdam, The Netherlands; 5 Department of Urology, The Third Medical Center, Chinese PLA General Hospital, Beijing, China

**Keywords:** Nerve growth factor, Perineural invasion, Neurotrophin pathway, Malignancy

## Abstract

**Background:**

As the first discovered member of the neurotrophin family, nerve growth factor (NGF) plays fundamental roles in peripheral sensory and sympathetic neuronal development and survival. Recent evidence reveals its tumour-promoting effects through increasing perineural invasion, which is correlated with poor clinical outcomes. The exact molecular mechanisms exhibit malignancy-specific differences and remain incompletely characterized.

**Main text:**

This review compares mechanistic insights and therapeutic advancements regarding NGF signalling in neurological/psychological disorders with discoveries in oncological contexts. Functioning as a dual biomarker for neural integrity and pathological progression, NGF primarily exerts its effects via an interaction with the high-affinity tyrosine kinase receptor. Both molecules are frequently overexpressed in malignant tissues. NGF orchestrates tissue regeneration and tumourigenesis through the activation of conserved neurotrophin pathways and downstream proliferative cascades, some of which participate in regulating the expression and secretion of NGF in turn. In practical applications, in addition to acting as an antiproliferative target, NGF could be utilized in psychological management, antinociceptive treatment, and wound healing.

**Conclusions:**

Systemic NGF-targeted therapies have significant articular and neurological toxicity, indicating the critical need for localized intervention strategies depending on the expression level of NGF and TrkA to balance antitumour efficacy with protective requirements for nerve structures and innervation.

## Introduction

Malignant tumours constitute one of the major diseases that endangers the health and life of people worldwide and remain to be addressed (Siegel et al. 2024). Surgery is the best option for cancer patients in the early stages, but postoperative recurrence is a Gordian knot (Reticker-Flynn and Engleman 2023). Currently, despite great progress in treating cancerous diseases, e.g., chemotherapy, radiotherapy, immunotherapy and targeted therapy, morbidity and mortality remain high due to tumour heterogeneity, resistance to comprehensive treatments, nonspecific clinical symptoms, and a delayed diagnosis (Setiawan et al. 2023). A high propensity for the metastasis of malignancies is the leading cause of cancer-related death and is difficult to address because of the characteristics of vascular invasion, lymph node metastasis, perineural invasion, etc. (Ohtsuka et al. 2002). An urgent need is to understand the molecular mechanisms of the pathogenesis and development of cancerous lesions to find new diagnostic and therapeutic approaches. One of the possible reasons is the dysfunctional tumour microenvironment (TME), which is composed of several cell types (e.g., immune cells and fibroblasts) and distinct expression patterns of protein subgroups (e.g., rearrangement of surface proteins and abnormal secretion of cytokines) (Yang et al. 2023; Kirchhammer et al. 2022).

Nerve growth factor (NGF) is the first discovered member of the neurotrophin family and promotes the growth and survival of peripheral sensory and sympathetic neurons. Additionally, as a significant cytokine, NGF functions to fine-tune the activation status of stromal cells and the proliferation and differentiation of epithelial cells and neural stem cells (Rocco et al. 2018). Abnormal NGF expression and secretion are detected in the body fluids of patients with neurological and psychological disorders, such as viral encephalitis, neurogenic bladder disease, schizophrenia, and Alzheimer’s disease (Turkmen et al. 2021; Rauch et al. 2022; Florentinus-Mefailoski et al. 2021; Homma et al. 2013). In addition to nervous system neoplasms, NGF is involved in the malignant transformation of other tissue cells, partly via nerve fibres and their secretory proteins near primary and metastatic cancerous lesions. Superfluous NGF in TME influences the biological behaviour of cancer cells (e.g., tropism and proliferation), which is called tumour neurotrophic activity (Banh et al. 2020). Additionally, nerve-derived NGF enhances perineural invasion (called PNI or axon-guidance activity) and ultimately results in the irritative and disabling symptoms of certain types of malignancies (Ricci et al. 2022). PNI was verified to be an unfavourable independent indicator of the survival of patients with malignancies such as pancreatic cancer, colorectal cancer (CRC) and gallbladder cancer (Newhook et al. 2023; Park et al. 2023; Pai et al. 2022). Therefore, the value of NGF is not restricted to its function as a neurological and psychological biomarker and is worthy of study for clinical application in oncology wards.

In this review, we focused on recent and previous mechanistic studies and clinical trials of NGF, especially the role of NGF in the crosstalk between nerve cells and malignant cells such as digestive system neoplasms. First, the molecular mechanisms of NGF and related signalling pathways are discussed. Second, achievements in research on NGF in neurological and psychological disorders are summarized. Next, we compared the updated findings of NGF in cancerous lesions to look for similarities and universalities. Finally, we reviewed and proposed possible future directions for the application of NGF.

## NGF and signalling pathways

### NGF overview

**Fig. 1:**
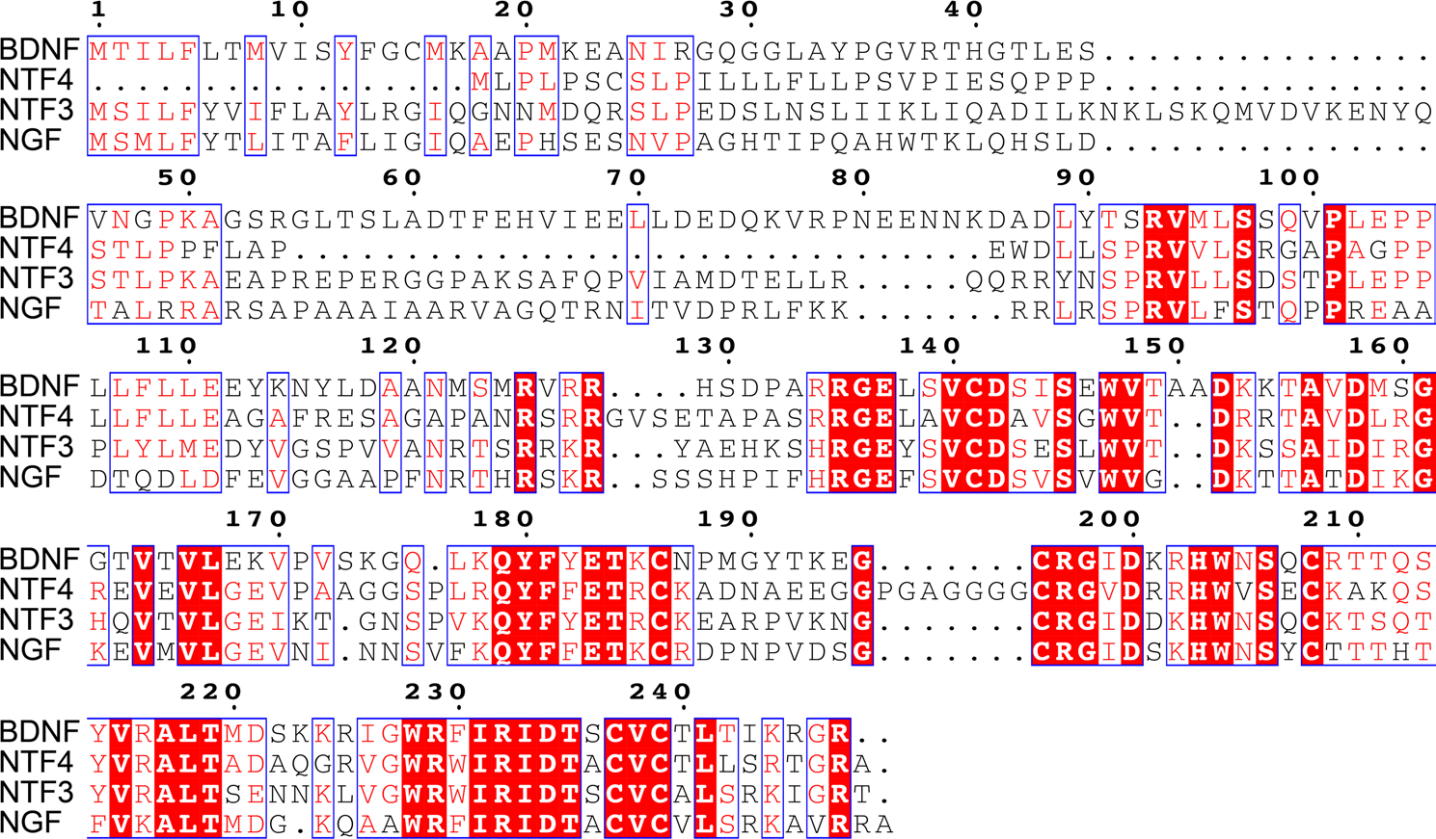
The result of protein sequence alignment by CLUSTALW and ESPript 3.0. The structure of neurotrophin family members were compared. The sequences were downloaded from the Uniprot database (https://www.uniprot.org/). Red, conservative sequence. Yellow, high similarity. White, low similarity

Neurotrophin family proteins are famous for their ability to stimulate the growth and differentiation of peripheral sensory and sympathetic neurons, thus promoting the healing, outgrowth and hypertrophic process of nerve fibres. Neurotrophin deprivation leads to the spontaneous apoptosis and degeneration of neurons (Bailly and Gao 2020; Kristiansen and Ham 2014). Among these family members, NGF was first discovered by Rita Levi-Montalcini and colleagues when they isolated tumour nucleoproteins from murine cells in 1954 (Chirchiglia et al. 2020). NGF is widely expressed in many tissues and is specifically synthesized and secreted by exocrine gland cells (Pingitore et al. 2016). The N-terminal domain of NGF reportedly functions as a whole protein structure in the presence of copper ions (Magrì et al. 2021). The other members of the neurotrophin family include brain-derived neurotrophic factor (BDNF, encoded by the *BDNF* gene), neurotrophin-3 (NT-3, encoded by the *NTF3* gene) and neurotrophin 4/5 (encoded by the *NTF4* gene), along with NGF, all of which possibly share structural and functional homologies and common ancestor genes (Rocco et al. 2018; Thompson et al. 1994; Robert and Gouet 2014). Discrete but common regions are found in the C-termini of the four proteins when the sequences are compared and analysed, providing evidence for similar physiological or pathological effects (red regions, Fig. 1). Therefore, in some retrospective clinical studies, these members have been analysed together to validate their diagnostic roles, such as in cholangiocarcinoma, COVID-19 infection and temporomandibular disorder (Westphalen et al. 2019; Demers-Mathieu et al. 2021; Jasim et al. 2020).

### NGF and its receptors

The biochemical precursor of the NGF protein is ProNGF, which is transcribed and encoded by the *NGF* gene. Before the synthesis of mature NGF, ProNGF is cleaved by furin and proconvertase in the intracellular environment, while secreted ProNGF is processed by plasmin and matrix metalloproteinases (MMPs) (Teng et al. 2010; Marsland et al. 2023). ProNGF has been confirmed to exert biological effects independent of mature NGF rather than functioning as a nonfunctional intermediate product. The evidence is described below. First, the distributions of NGF and its precursors are different in human tissues. For example, the ProNGF level is far higher than NGF in brain tissue (Fahnestock et al. 2001). Second, due to protein metabolic processes (e.g., the removal of the signal peptide), the differences in molecular structure between NGF and ProNGF are considerable. Third, the normal function of NGF depends on the expression level and binding affinity of receptor proteins. NGF preferentially binds to neurotrophic tropomyosin receptor kinase A (TrkA, encoded by the *NTRK1* gene), whereas the precursor form has a greater affinity for p75 neurotrophin R (p75NTR, encoded by the *NGFR* gene) and Sortilin 1 (encoded by the *SORT1* gene) (Morisse et al. 2023).

The activation level and duration of NGF signalling are strictly regulated by the expression level of not only different forms of neurotrophins but also various types of NGF receptors. Both ligands and receptors are subjected to fine-tuning by protein metabolic pathways. First, the overall direction of the NGF-mediated neurotrophin signalling pathway is determined by its interaction with different receptor proteins. TrkA, the high-affinity receptor of NGF, is composed of five extracellular domains (ECDs), a transmembrane domain (TM), and an intracellular domain (ICD) (Conroy and Coulson 2022). The ECDs are implicated in capturing and binding ligands. The intracellular segment contains a kinase domain, which is a conserved structural feature of the tyrosine kinase family. The NGF-TrkA interaction induces TrkA multimerization, increasing the phosphorylation level and tyrosine kinase activity of TrkA. Activated TrkA subsequently phosphorylates downstream substrate proteins to trigger related signalling pathways (Regua et al. 2021). Another receptor, p75NTR, is a member of the tumour necrosis factor (TNF) receptor superfamily. p75NTR contains four cysteine-rich domains (CRDs) and glycosylation sites in the ECD, a linker segment in the TM, and a p75 death domain in the ICD (Han et al. 2023). Unlike TrkA, p75NTR lacks tyrosine kinase structures and is characterized by nonspecific binding to nearly all the neurotrophins and corresponding precursors, although the affinity varies considerably (Lu et al. 2005). Sortilin 1, a member of the Vps10p-domain receptor family, is a coreceptor of p75NTR and contributes to the ProNGF-p75NTR interaction in proapoptotic pathways. The other two coreceptors are the Nogo receptor (encoded by the *RTN4R* gene) and LINGO-1 receptor (encoded by the *LINGO1* gene). Sortilin 1 was reported to bind only to the precursor forms of neurotrophins (Seidah et al. 1996).

In some situations, the functions of TrkA and p75NTR were antagonistic (Volosin et al. 2006; Longo and Massa 2013; Reitz et al. 2024). In prurigo nodularis lesions, TrkA is upregulated and p75NTR is downregulated (Zhong et al. 2019). Activated TrkA is recognized as a promoter of cell proliferation, differentiation, nerve outgrowth and angiogenesis (Conroy and Coulson 2022; Campos et al. 2007; Vera et al. 2014). In addition, TrkA activated by NGF inhibits spontaneous apoptosis, whereas p75NTR activated by ProNGF exerts the opposite effects. High expression levels of TrkA, rather than p75NTR, are correlated with abdominal pain and PNI (Dang et al. 2006; Ma et al. 2008). Accordingly, TrkA is regarded as an oncoprotein in most instances, which is consistent with the discovery of the fusion *NTRK1* oncogene and the role of wild-type TrkA as a potential oncogenic driver. NTRK inhibitors, e.g., entrectinib and larotrectinib, can be applied to treat cancerous lesions in which abnormal expression of the *NTRK* gene is promoted by gene fusion (Regua et al. 2021; Jiang et al. 2021).

The relationship between NGF and receptor proteins is worth studying. In the molecular structure of NGF, certain amino acid residues (e.g., His4, Arg9 and Glu11) stabilize the interaction between NGF and its receptors (Vittorio et al. 2022). Even though the activated status of two NGF receptors orchestrates diverse biological processes, some exceptions have been reported, which cannot be explained by previous findings. When NGF binds to low-affinity p75NTR for various reasons, its overall biological effect promotes cell survival. Given that p75NTR is upregulated in certain malignancies, e.g., seminomas, determining the exact role of p75NTR in tumour progression is difficult (Soligo et al. 2019; Perri et al. 2021). When melanoma cells are treated with sulforaphane, NGF application reverses chemical-mediated apoptosis induction and migration inhibition (Arcidiacono et al. 2018). In neuroepithelioma cells, NGF prompts spontaneous apoptosis in a heterogeneous nuclear ribonucleoprotein (hnRNP)-dependent manner (Jung et al. 2016). The interaction between ProNGF and p75NTR leads to the differentiation of satellite cell-derived myoblasts into myotube structures, yet cell proliferation is not significantly influenced (Pallottini et al. 2020).

Based on the evidence mentioned above, the regulatory network between NGF/ProNGF and TrkA/p75NTR can be inferred to be not simple, dualistic or static but rather complex, interrelated and dynamic. First, the distributions of NGF and receptor proteins depend on the cell context and functional status, which can be reflected by the NGF/ProNGF ratio and TrkA/p75NTR ratio according to protein kinetics. MMP overactivation, such as MMP7 dysfunction, augments the transformation from ProNGF to NGF (Kucharczyk et al. 2016; Mossa et al. 2021). MMP9 participates in the degradation of mature NGF. The activity of MMP9 can be further diminished by TIMP metallopeptidase inhibitor 1 (TIMP1) and promoted by nitric oxide and prostaglandin E synthase 1 (PGE1) (Pentz et al. 2021; Ridnour et al. 2007; Khan et al. 2012). The overexpression of certain E3 ubiquitin ligases, such as TNF receptor-associated factor 4 (TRAF4), results in the degradation of NGF receptors (Singh et al. 2018). Second, the mature and precursor forms of NGF affect the protein levels of the corresponding receptors. Exogenous NGF aggravates the cleavage of p75NTR by activating ADAM metallopeptidase domain 17 (ADAM17) in ovarian cells and γ-secretase in brain cells (Garrido et al. 2022; Forsyth et al. 2014). Third, the specific NGF-TrkA interaction is enhanced by p75NTR (e.g., avoidance of alternative pathway activation by other neurotrophin members), which is consistent with the coexpression and common distribution of these two receptors (Hempstead et al. 1991). Fourth, exogenous and endogenous factors, such as the phosphatase SHP-1, other neurotrophin members, different variants of NGF and blue light illumination, can influence the functions of TrkA and p75NTR in an NGF- and proNGF-independent manner. Polymorphisms in genes encoding NGF receptors, especially in malignant cells, also complicate the understanding of the ambivalent phenomenon of ligand-receptor binding (Soligo et al. 2019; Yang et al. 2022; Zhao et al. 2020; Duan et al. 2018; Montano 2009).

### NGF-related signalling pathways

Once TrkA interacts with NGF, tyrosine kinase regions are activated, triggering a phosphorylation cascade beginning with second messengers and activating the neurotrophin signalling pathway, which is composed of multiple downstream proliferation-associated pathways and a complex regulatory network. The overall biological effect promotes nerve growth and improves metabolic functions, e.g., maintaining axoplasmic transport and action potential (Peach et al. 2024). The substrates include extracellular signal-regulated kinase 1/2 (ERK1/2) and the serine/threonine kinase AKT, which mediate the mitogen-activated protein kinase (MAPK)/ERK pathway and phosphoinositide-3 Kinase (PI3K)/AKT pathway, respectively (Sanchez-Vega et al. 2018; Polara et al. 2024; Wang et al. 2021a, b; Sementino et al. 2024). Both pathways participate in maintaining the proliferation, angiogenesis, migration and invasion of malignant cells by stimulating the expression of related downstream genes, such as vascular endothelial growth factor (VEGF), MMPs, CD133, zinc finger E-box binding homeobox 1 (ZEB1) and MYCN proto-oncogene bHLH transcription factor (MYCN) (Trejo et al. 2012; Romon et al. 2010; Han et al. 2022; Chen et al. 2021; Xin et al. 2016; Lei et al. 2022). The MAPK/ERK pathway is reportedly activated by ectopic NGF secretion in neuroblastoma, breast cancer, colon cancer and pancreatic ductal adenocarcinoma (PDAC) (Romon et al. 2010; Han et al. 2022; Xin et al. 2016; Lei et al. 2022; Lebedev et al. 2021). In contrast, activation of the PI3K/AKT pathway has been observed in PDAC, oral and salivary gland tumours, prostate cancer (PC) and glioma (Chen et al. 2021; Jiang et al. 2020a, b; Meco et al. 2019; Alkhadar et al. 2020). Additionally, activation of the PI3K/AKT pathway is accompanied by an antiapoptotic effect. NGF deprivation leads to neuronal apoptosis, while NGF overexpression protects salivary gland cells from apoptosis, probably by dephosphorylating the c-Jun N-terminal kinase (JNK) (Akhter et al. 2018; Li et al. 2021). The PI3K/AKT pathway also enables the activation of downstream pathways, such as the Wnt pathway, by dephosphorylating β-catenin to maintain its stability (Garrido et al. 2020a, b; Tao et al. 2013). NGF/TrkA can also directly activate the Wnt pathway, enhancing the proliferation of gastric epithelial cells and osteocytes (Hayakawa et al. 2017; Tomlinson et al. 2017). In addition to promoting proliferation, dedifferentiated cells are forced to differentiate upon NGF application (e.g., the rat adrenal pheochromocytoma cell line PC12, which is commonly utilized as a nerve cell model) (Mouri and Sako 2013; Linciano et al. 2023).

**Fig. 2:**
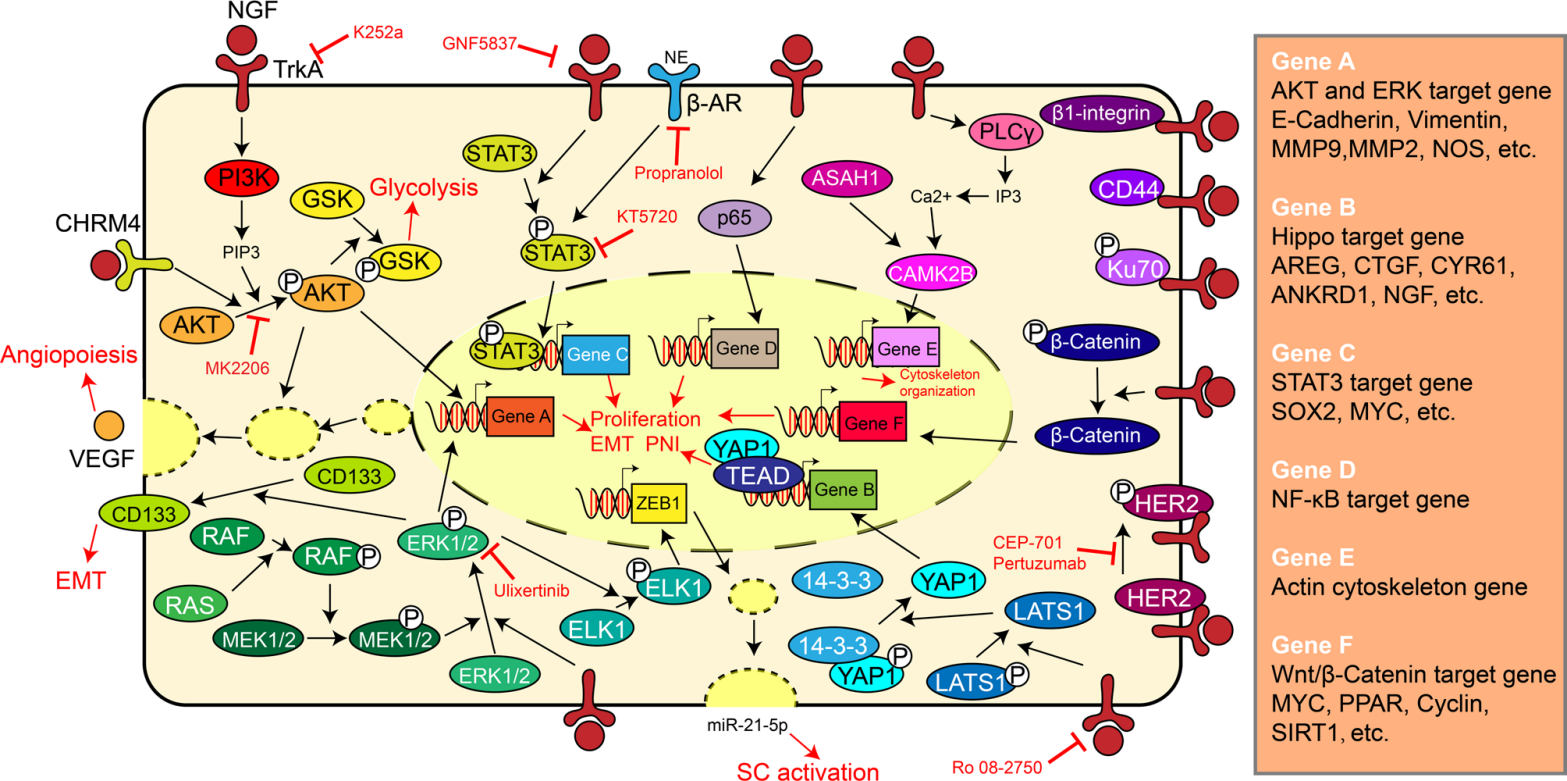
Summary of the signalling pathways activated by NGF. The inhibitors for different steps and the biological effects are marked in red. Arrow, promotion. Flat arrow, inhibition

Inflammatory pathways, such as the NF-κB pathway and Janus kinase (JAK)-signal transducer and activator of transcription (STAT) pathway, are implicated in the regulation of malignant behaviours. The phosphorylation level of STAT3, a key mediator of the JAK-STAT pathway, is increased by NGF because STAT3 serves as a substrate of TrkA (Regua et al. 2021). During the development of neurologic tumours or PNI in some nonneurologic tumours, the NGF/TrkA/STAT3 axis significantly facilitates the epithelial‒mesenchymal transition (EMT) of malignant cells (Meco et al. 2019; Lin et al. 2020; Guo et al. 2013). NGF is also involved in the activation of other pathways, e.g., the Hippo and phospholipase signalling pathways. which is summarized in Fig. 2 (Tsai et al. 2018; Wong et al. 2019). Additionally, some studies reported that NGF stimulates the translocation or expression of the phosphorylated p65 protein and activates the NF-κB pathway to play protective roles, e.g., exert antiapoptotic and proangiogenic effects on cells exposed to cytotoxic agents, mainly through NGF/p75NTR instead of the NGF/TrkA complex (Esposito et al. 2021; Naderi et al. 2007; Jung et al. 2022). However, a study of chondrocytes indicated that NGF/p75NTR-mediated NF-κB activation aggravated the apoptosis induced by an acidic environment (Wei et al. 2020). NGF-related biological processes can be targeted by NF-κB pathway inhibitors, e.g., triptolide (Nomura et al. 2016). In contrast, many studies have reported the concomitant upregulation of NF-κB domains and NGF, but the intrinsic connections and functional relevance between these factors have not been discussed in detail (Meyers et al. 2020; Salama and Elgohary 2023; Yin et al. 2023; Lin et al. 2024a, b). Some evidence has shown that NGF/TrkA signalling has the opposite effect on the NF-κB pathway. Mechanistically, NGF blocks the expression of inflammatory cytokines (e.g., IL-1β, TNF-α, IL-6, and IL-8) and scavenges reactive oxygen species (ROS) by inhibiting Toll-like receptors (TLRs) in corneal epithelial cells and monocytes (Chen et al. 2019; Prencipe et al. 2014; Wang et al. 2023). When cerebral trauma occurs, NGF decreases the acute induction of IL-1β expression and impedes the ability of NF-κB to bind to DNA (Lv et al. 2014). NGF also inhibits the phosphorylation of IκB kinase and impairs the transport of NF-κB into the nucleus (Chacón and Rodríguez-Tébar 2012). NGF also reorganizes vascular cell adhesion molecule-1 (VCAM-1). When monocytes bind to VCAM-1, polarization to the M2 phenotype occurs, which contributes to the secretion of anti-inflammatory cytokines, e.g., IL-10 (Prencipe et al. 2014; Lin et al. 2024a, b). The exact role of NGF, whether it is the primary cause of inflammation or the secondary factor involved in neurotrophy to withstand inflammatory damage, could be inferred to determine the synergistic or antagonistic effects on NF-κB and other proinflammatory pathways. From another perspective, the regulatory mechanism of NGF expression might be fundamental for switching the destructive role to a protective role (Zeng et al. 2021; He et al. 2018; Dai et al. 2020).

### Factors influencing the function and secretion of NGF

As a pluripotent cytokine, NGF is often induced by noxious stimuli, e.g., cigarette exposure to bronchial epithelial cells and exposure to ROS during oral mucosa carcinoma progression, leading to airway inflammation and nociceptive nerve hyperfunction (Zhang et al. 2022a, b; Stabile et al. 2018). PC12 cells are commonly used to evaluate NGF-mediated cell behaviours and screen natural or synthesized compounds that interfere with the function of NGF (Wiatrak et al. 2020). NGF-induced neurite outgrowth is inhibited by scabronine M (extracted from Strobilurus tenacellus), Rhosin (a predicted Rho GTPase inhibitor), and SUTAF-027 (a chemically synthesized β-methoxyacrylate antibiotic) (Bailly and Gao 2020; Nagahara et al. 2012; Shang et al. 2012). Oxaliplatin not only inhibits NGF-mediated nerve growth but also reduces the NGF-mediated increase in the intracellular calcium concentration, which is essential for maintaining neural excitation and cytoskeletal rearrangement (Takeshita et al. 2011). However, recent pharmacological studies have seldom focused on the common molecular mechanism between PC12 cells and other tissue types, which limits the extension of theoretical models to translational studies of antiproliferative agents.

**Fig. 3:**
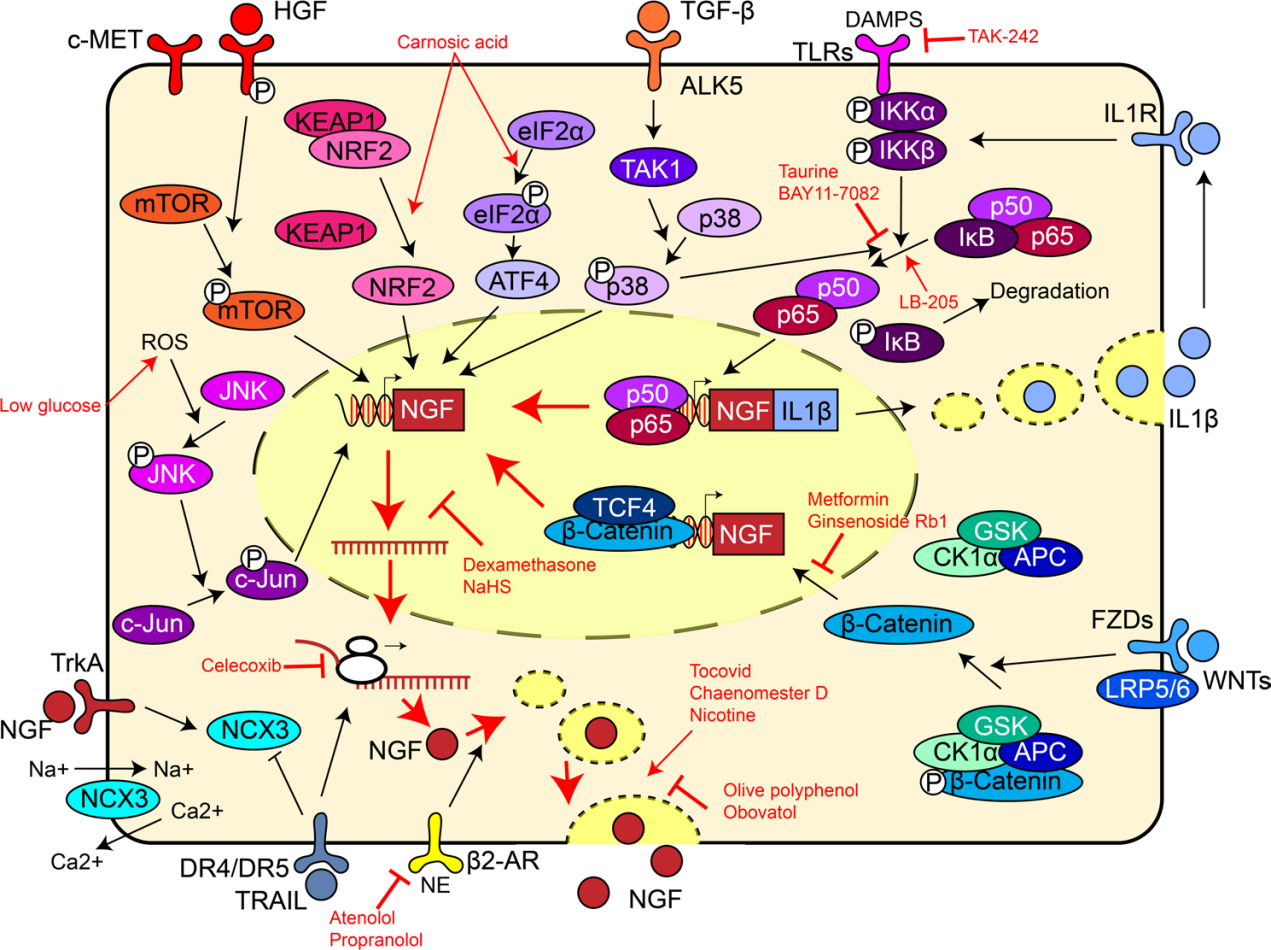
Summary of the signalling pathways that affect the expression and secretion of NGF. The inhibitors for different steps and the biological effects are marked in red. Arrow, promotion. Flat arrow, inhibition

The synthesis and secretion of NGF are strictly regulated in cancerous lesions, e.g., intratumoral parenchyma cells and various cell subgroups in the carcinogenic microenvironment. For example, norepinephrine (NE), an important sympathetic adrenergic neurotransmitter, stimulates NGF secretion from triple-negative breast cancer (TRBC) cells by activating the MAPK/ERK pathway (Jin et al. 2023). Hepatocyte growth factor (HGF) induces NGF production in PDAC cells via similar downstream mechanisms (Qin et al. 2022). Confusingly, different effects on the neurotrophin pathway were observed upon the administration of several compounds, most of which serve as anti-inflammatory agents or components of antiphlogistic herbal preparations. Celecoxib, a cyclooxygenase-2 (COX-2) inhibitor, could downregulate NGF, relieving chronic pain due to head and neck squamous cell carcinoma (HNSCC) (Yang et al. 2015a, b). However, the viability of HNSCC cells is not affected. In contrast, chaenomester D, a natural anti-inflammatory oxylipin, not only promotes NGF secretion but also inhibits the proliferation of non-small cell lung cancer (NSCLC) cells (Lee et al. 2023). Such contradictions were also observed in normal tissues. Obovatol induces NGF release from nerve cells and increases neurite outgrowth (Lee et al. 2010). However, when dexamethasone is applied to lens epithelial cells, NGF expression is downregulated, and both proliferation and migration are impaired (Hah et al. 2020). Mechanistically, certain pathways, e.g., the Wnt and NF-κB pathways, could upregulate NGF at the transcriptional level. A study of monocytes revealed that the NGF/TrkA pathway is involved in negative feedback during the inflammatory response, as NF-κB-mediated NGF expression reduces the level of phosphorylated IκB, supporting the anti-inflammatory role of the NGF/TrkA complex (Prencipe et al. 2014). Neurotransmitters, e.g., NE and acetylcholine (ACh), stimulate NGF secretion in epithelial tissue. In response, extracellular NGF increases the secretion of neurotransmitters, e.g., 5-hydroxytryptamine, in lung cancer, supporting the connection between nerve cells and malignant transformation (Jin et al. 2023; Zheng et al. 2024a, b). The related molecular mechanisms are summarized in Fig. 3 (Lv et al. 2014; Kobayashi et al. 2024; Nicoló et al. 2013; Moniaga et al. 2020; Mimura et al. 2019).

In conclusion, NGF enhances nerve growth, likely by activating inflammation-related pathways. Nevertheless, this phenomenon is not always applicable to differentiated nonneural tissues, such as HNSCC, NSCLC, and eye tissue. The regulation of the expression patterns of NGF and proinflammatory cytokines by the same stimulus differed. Distinct cellular responses to agents with similar pharmacological actions have been observed in different tissues. Although the molecular structures of these compounds differ, the overall anti-inflammatory effects of these compounds have been verified in clinical practice and empirical medicine, which reflects the intricate relationship between NGF and the pathogenesis of inflammatory-associated diseases. In a broader sense, we cannot simply define the exact impact of pathological changes in NGF levels in body fluid or the perilesional environment on general homeostasis. Based on the existing evidence, the reasons listed below have been proposed. First, the interactions between bioactive compounds and NGF are unknown. This question is further confused by whether NGF upregulation is the cause or result of inflammation occurrence and how these compounds intervene in the actions of key regulators. Second, the expression level of NGF and the distribution of NGF receptors in human tissues are worth studying. The NGF/p75NTR interaction contributes to the activation of inflammatory pathways, whereas the NGF/TrkA interaction inhibits the development of inflammation. Third, the cells in the local environment serves as mediators and regulators of the neurotrophin signalling pathway. For example, the serum NGF level is positively correlated with the peripheral eosinophil count (Ma et al. 2021). Given the overall effect of NGF in mediating healing, outgrowth and hypertrophic process of nerve tissue, the relationship between NGF and inflammation may hinge on different stages of inflammation development and the time-dependent expression patterns of proinflammatory cytokines or mediators.

## NGF: a neurological and psychological biomarker

### NGF and nerve-associated diseases

Neurodegenerative diseases are a group of clinically heterogeneous and complex age-related disorders that are characterized by the progressive degeneration or death of neurons in the central or peripheral nervous system (Ricci 2024). Alzheimer’s disease (AD) is one of the most studied neurodegenerative diseases, and the main symptom is cognitive decline (Busquets et al. 2021). The main pathogenic features of AD are β-amyloid (Aβ) plaques and neurofibrillary tangles (Goure et al. 2014). The Aβ protein is derived from the cleavage product of amyloid precursor protein (APP). Many studies have shown that NGF is downregulated in the brain tissue and cerebrospinal fluid (CSF) of patients with AD, which is also observed in patients with Parkinson’s disease, another neurodegenerative disease, indicating that decreased NGF levels might be associated with nerve tissue hypofunction and degradation (Santaella et al. 2020; Williams et al. 2006). Another piece of evidence is that NGF delivery via gene therapy or special delivery through the blood‒brain barrier (BBB) can attenuate neuronal death (Florentinus-Mefailoski et al. 2021; Tuszynski et al. 2015). The likely molecular mechanism is that NGF promotes the normal interaction between APP and TrkA by disrupting APP phosphorylation at Thr668 in hippocampal neurons to prevent pathological Aβ aggregation and the overaction of proapoptotic factors, e.g., Bcl-2-modifying factor (Bmf) (Akhter et al. 2018; Triaca et al. 2016; Matrone et al. 2008).

Sometimes, the overaction of organs or tissues with abundant innervation is also accompanied by NGF overexpression. Nerve lesions are one of the causes of bladder disease, which manifests as paraesthesia (e.g., odynuria) and paruria (e.g., frequent and urgent micturition). The urinary NGF (uNGF) level is used as a diagnostic biomarker and is increased in adult patients with interstitial cystitis and paediatric patients with nocturnal enuresis, along with other neurotrophin members and chemokines, e.g., CXCL9 (Homma et al. 2013; Morizawa et al. 2019). The uNGF/creatinine ratio has been used to assess the severity of childhood dysfunctional voiding (Ergin et al. 2016). When autologous platelet‑rich plasma is injected intravesically to treat refractory interstitial cystitis, the uNGF level decreases significantly and bladder irritation is relieved (Jiang et al. 2020a, b). Strangely, if surgery is performed on female patients with overactive bladders, uNGF is upregulated until 3 months postsurgery, indicating different NGF expression patterns in iatrogenic injuries and tissue repair (Wu et al. 2021). Even though the source of uNGF remains unknown, in view of the overactive irritative manifestation of the bladder, inflamed transitional epithelial tissue and infiltrated immune cells might secrete uNGF, which results in the hyperactivity of the nerve fibres supplying the bladder. This mechanism could be applied to other inflammatory diseases that arouse hyperesthesia or other forms of discomfort.

Common causes of nerve injury include ischaemic injury and infection. The serum NGF level in certain patients with acute ischaemic stroke is higher than that in healthy individuals. Continuous NGF upregulation in the postinjury stage indicates improved recovery and functional outcomes (Luan et al. 2019). Similarly, pseudorabies virus infection downregulates the NGF mRNA level. The administration of the antiviral agent resveratrol alleviates the neuronal apoptosis by reversing the inhibitory effect, suppressing M1 microglial polarization and activating M2 microglial polarization (Chen et al. 2023). Some viral infections, e.g., human T-lymphotropic virus 1, can stimulate NGF secretion into the CSF (Freitas et al. 2022). In general, NGF upregulation after nerve injury is a sign of nerve regeneration, even though this compensatory reaction is sometimes deleterious (e.g., the role of NGF as a biomarker indicating hepatocellular carcinoma development after anti-hepatitis C treatment) (Owusu Sekyere et al. 2021). When paclitaxel-induced peripheral neuropathy is treated with N-acetylcysteine or metformin, increased serum NGF levels are observed, along with the remission of paraesthesia and sensory loss (Khalefa et al. 2020; Bakry et al. 2023). In contrast, NGF levels decrease under the following conditions. First, proteins participating in alternative signalling pathways might affect NGF expression, e.g., anti-nucleoprotein activity via SARS-CoV-2 and anti-inflammatory interleukin activity via the pseudorabies virus (Chen et al. 2023; Usai et al. 2021). Second, NGF levels fail to compensate for extreme circumstances, e.g., severe emerging encephalitis caused by human Borna disease virus 1 and impaired immunity caused by human immunodeficiency virus (Rauch et al. 2022; Asahchop et al. 2018; Sirico et al. 2023).

### NGF and psychological disorders

NGF dysregulation is also observed in individuals with psychological diseases. Schizophrenia is a chronic and progressive mental disorder caused by neurodevelopmental and neurodegenerative factors, indicating potential pathogenic processes mediated by NGF (Martinez-Cengotitabengoa et al. 2016). NGF is detected in serum samples, and in most studies, the NGF levels in patients with schizophrenia are lower than that in healthy individuals. A study in Turkey enrolled hospitalized patients with schizophrenia (Turkmen et al. 2021). Another study in China selected specific individuals, i.e., unmedicated patients with first-episode schizophrenia presenting positive symptoms, and reached similar conclusions (Qin et al. 2022). When antischizophrenic drugs, e.g., olanzapine and lurasidone, are administered to drug-naive patients, NGF levels along with other neurotrophic factors, e.g., BDNF and NT-3, are increased, which supports the essential role of neurodegenerative disorders in schizophrenia etiopathogenesis (Jena et al. 2019). Additionally, the onset of negative symptoms is negatively correlated with the serum NGF level according to a multicentre longitudinal trial, indicating the value of NGF as a potential predictor of the long-term severity and course of schizophrenia (Martínez-Pinteño et al. 2022). A similar phenomenon is observed in patients with depression. NGF levels in blood samples are decreased in patients with major depressive disorder (MDD) (Shi et al. 2020). Elevated NGF levels are observed under certain circumstances, e.g., sertraline treatment of patients with mild and moderate depression without somatic syndrome (Mishra et al. 2019).

Neuroplasticity is the ability of the nervous system to adapt its structural and functional properties in response to internal and external stimuli. According to the neurodegeneration theory, in patients with schizophrenia and depression disorders, the molecular mechanisms of neuroplasticity sometimes manifest as abnormal functions due to NGF deficiency. The NGF-TrkA interaction promotes cytoskeletal rearrangement and neurite outgrowth by activating phospholipase C-γ (PLC-γ), leading to the synthesis of inositol triphosphate (IP3), the release of Ca2+, and the activation of calcium/calmodulin-dependent protein kinase II beta (CAMK2B). Schizophrenia is exacerbated by the inhibition of this pathway (Su et al. 2021; Itoh et al. 2011). In certain regions of the brain in a depression model, e.g., the prefrontal cortex and hippocampus, NGF downregulation is accompanied by the suppression of the cAMP/cAMP response element binding protein (CREB), PI3K/AKT, and MAPK/ERK pathways (Li et al. 2023; Xia et al. 2023). However, in another dexamethasone-induced depression model, rosiglitazone exerts antidepressant effects via NGF activation and decreases the levels of phosphorylated AKT and mechanistic target of rapamycin kinase (mTOR), which conflicts with the conclusions above (Alhaddad et al. 2023). Possible reasons might be the lack of human samples, the use of various methods for modelling, and heterogeneous pathogenic factors for neurodegenerative diseases, which require further studies.

### NGF and pain

Pain is defined as “an unpleasant sensory and emotional experience associated with actual or potential tissue damage, or described in terms of such damage” by the International Association for the Study of Pain (Testa et al. 2021). Pain is a subjective experience that largely depends on how the brain processes damage signals from peripheral nerve fibres rather than simply the form, severity, and duration of the stimulus that triggers nociception (Schmelz et al. 2019). NGF is involved in pain genesis and transmission because of its pronociceptive function. Local NGF injection into the right radialis forearm muscle leads to persistent lateral elbow pain (Summers et al. 2019). The serum NGF level is positively correlated with the frequency of episodic migraine (Mozafarihashjin et al. 2022). Microcosmically, NGF mainly binds to TrkA distributed on the surface of small myelinated and unmyelinated fibres, resulting in the phosphorylation and sensitization of nociceptors, e.g., transient receptor potential cation channel 1 (TRPV1) and acid-sensing ion channel 3 (ASIC3) (Velasco et al. 2017; Mantyh 2019; Arendt-Tranholm et al. 2024). When trauma occurs, NGF can be released from damaged tissues, e.g., injured bones, leading to local discomfort or acute pain (Mantyh 2019; Kan et al. 2024). Extracellular NGF also interacts with TrkA on immune cells, and the latter migrate to nerve tissue and secrete inflammatory mediators (e.g., PG and histamine), further aggravating acute pain (Schmelz et al. 2019; Subramanian et al. 2021; Da Vitoria Lobo et al. 2024). Additionally, long-term NGF stimulation drives the sprouting of peripheral and central sensory neurons, such as neurons in the dorsal root ganglion (DRG) and dorsal horn, causing chronic pain and acute attacks (Barker et al. 2020; Liu et al. 2024; Wang et al. 2022; Zuo et al. 2024). When NGF is targeted by neutralizing antibodies (anti-NGF mAbs), a decrease in TRPV1 levels is observed. The functional status of NGF also influences pain perception. Patients with hereditary sensory and autonomic neuropathies (HSANs) are characterized by painlessness and abnormal behaviour. NGF mutation is considered a possible cause. R221W, V232fs, and R121W mutations inhibit the conversion of ProNGF to NGF and impair its pain-regulating function, while the R221W mutation maintains neurotrophic properties (Testa et al. 2021). The global somatic mutation of NGF might also impair the normal release of NGF from stromal cells, e.g., fibroblasts, resulting in structural deficits in intraepidermal nerve fibres (Yang et al. 2020).

## NGF and cancerous diseases

### NGF and nervous system neoplasms

Malignancies in the central nervous system (CNS) account for approximately 1.35% of all malignant tumours and 3% of cancer-related deaths. Among these cases, nearly 1/3 are primary brain neoplasms (Gritsch et al. 2022). Glioma is the most common and difficult-to-treat CNS malignancy originating from intracephalic gliocytes. The glioblastoma multiforme (GBM) type is the most aggressive subgroup in adults (Marsland et al. 2023; Brown et al. 2008). In contrast, neuroblastoma (NB) originates from extracranial neuroblast cells and is common in children, resulting in approximately 15% of paediatric cancer-related deaths (Lebedev et al. 2022). Compared with normal nerve tissue, malignant cells seem to preserve some biological features, such as sensitivity to neurotrophic factors. The expression level of NGF in glioma is nearly threefold higher than that in the normal brain (Brown et al. 2008). NGF promotes the growth, proliferation, aggressiveness, and angiogenesis of glioma cells in an α9β1 integrin-dependent manner. Pro-oncogenic signals, e.g., MAPK/ERK signals, are subsequently activated after the formation of the NGF-integrin complex (Brown et al. 2008; Walsh et al. 2012). The JAK/STAT and PI3K/AKT pathways can also be activated by NGF in the context of glioma and neuroblastoma (Meco et al. 2019; Benedetto et al. 2019). However, except for Benedetto et al., related studies have seldom mentioned the exact role of TrkA in NGF-induced activation of the proliferation pathway (Benedetto et al. 2019). This role could be partly explained by the unique expression patterns and functions of ProNGF and p75NTR. Unlike in other types of tumours, ProNGF is upregulated in GBM and stimulates the proliferation of parenchyma cells (Marsland et al. 2023). A similar phenomenon can also be observed in PDAC, and upregulated NGF rescues tumour cells from programmed death (Xu et al. 2019). Mature p75NTR cleavage and subsequent release of the ICD domain are mediated by α- and γ-secretases, which are commonly found in glioma lesions with increased invasive and proliferative abilities (Forsyth et al. 2014). Eventually, pro-oncogenic pathways are activated. Intriguingly, in benign and malignant gliocytes, pro-oncogenic signals contribute to the production of NGF, indicating potential positive feedback in accelerating glioma progression (Guo et al. 2020; Tseng et al. 2014). Functionally, NGF upregulation and pro-oncogenic pathway activation are conducive to rescuing glioma and NB cells from pro-apoptotic factors, such as tumour necrosis factor-related apoptosis-inducing ligand (TRAIL) and kinase inhibitors, e.g., imatinib, dasatinib, crizotinib, cabozantinib, and axitinib (Lebedev et al. 2021, 2022; Benedetto et al. 2019).

### NGF and male malignancy

PC is the most commonly diagnosed male malignancy and a leading cause of the cancer-related death of men in the Western population (Gu et al. 2024; Festuccia et al. 2009). One of the views of the aetiology of PC is an aberrant hormonal microenvironment, which results in androgen-dependent, inflammatory, and proliferative but noninvasive precancerous conditions before PC formation (Festuccia et al. 2009). Both normal prostatic tissues and PCs are capable of secreting NGF, yet their sensitivity is determined by the levels of TrkA and p75NTR, which induce mitogenesis and apoptosis, respectively (Donato et al. 2023a, b; Liu et al. 2021a, b). Physiologically, smooth muscle stromal cells lack NGF receptors, whereas prostate epithelial cells express both receptors (Rende et al. 2010; Anagnostopoulou et al. 2013). As PC develops and progresses, even though PC expresses both receptors, TrkA and NGF are upregulated, whereas p75NTR expression is gradually diminished, along with increased cell viability and invasion (Anagnostopoulou et al. 2013; Donato et al. 2019; Baspinar et al. 2017). Ultimately, p75NTR expression is almost absent in metastatic lesions of PC (Donato et al. 2023a, b),. Moreover, the aggression of PC cells might be aroused by NGF derived from perifocal cells, e.g., coculture with cancer-associated fibroblasts (CAFs) upregulates Yes-associated protein 1 (YAP1) in PC cells, which recruits TEA domain family member 1 (TEAD1) to the NGF promoter, sustaining NGF secretion and the perineural invasion of PC cells (Shen et al. 2022). In this context, targeting NGF with natural antibodies significantly inhibits PC metastasis in vitro (Warrington and Lewis 2011).

PC can be divided into androgen-dependent and androgen-resistant types based on their dissimilar responses to androgens. Androgen-dependent PCs are sensitive to androgen deprivation therapy (ADT), regardless of whether they directly target the androgen receptor (AR) or whether secondary decreases in androgen levels are induced by gonadotropin-releasing hormone (GnRH) analogues (Ong et al. 2024). Once stimulated by NGF, TrkA and AR could be assembled into a corresponding protein complex, activating proliferation pathways and escaping from apoptosis, suggesting that the communication between neurotrophins and the androgen pathway is cross-activated by each other (Donato et al. 2015, 2018). The synergistic effects of NGF and dehydroepiandrosterone (DHEA) on the upregulation and phosphorylation of TrkA has been documented, where testosterone blocks the above biological process and exacerbates PC cell apoptosis (Anagnostopoulou et al. 2013). This conclusion applies equally to the TrkA–human epidermal growth factor receptor 2 (HER2) complex (Festuccia et al. 2009). TrkA kinase activity can also be provoked by TRAF4-mediated K27- and K29-linked ubiquitination (Singh et al. 2018). As PC progresses, the expression of NGF and TrkA gradually increases, even robustly enough to replace the androgen-AR axis, which might explain the androgen resistance phenomenon observed upon first examination or after continuous ADT (Donato et al. 2019). ADT-induced neuroendocrine differentiation (NEPC) is also a challenge for PC treatment, resulting in an aggressive phenotype and poor outcomes. NGF binds to the cholinergic receptor muscarinic 4 (CHRM4) and activates the N-MYC-related pathway, which is the key causative factor for NEPC. ADT induces the activation of zinc finger and BTB domain containing 46 (ZBTB46) and maintains the excessive transcription and secretion of NGF (Lei et al. 2022).

### NGF and malignancies in females

BC is the most frequently diagnosed female malignancy worldwide and is also the leading cause of cancer-related death in women (Heer et al. 2020). Most BC cases are characterized by abnormal activation of the hormone signalling pathway. Accordingly, excess expression of hormone receptors, e.g., oestrogen receptor (ER) and progesterone receptor (PR), occurs in approximately 70% of BCs (Burstein et al. 2014). Approximately 15-20% of BC cases are TNBC, which is negative for ER, negative for PR and has no HER2 overexpression, and is accompanied by early lymphatic metastasis and a poor prognosis (Zheng et al. 2024a, b). Endocrine therapy, e.g., aromatase inhibitors, is the standard treatment for hormone receptor-positive BC. Letrozole was found to inhibit the secretion of NGF by fibroblasts and reverse the promotion of BC cell invasion (Li et al. 2016). NGF and TrkA are overexpressed in BC tissue compared with normal control tissue. High serum levels of NGF indicate poor outcomes for BC patients (Jung et al. 2021; Adriaenssens et al. 2008). The probable reason is the positive correlation between NGF and BC aggressiveness. First, NGF is involved in direct communications between BC cells and nerves, contributing to the PNI of BC cells. Histologically, isolated nerve fibres are frequently found in invasive ductal carcinoma or lymph node-positive tumours (Jung et al. 2021). Certain neurotransmitters, e.g., NE from the sympathetic nervous system, facilitate NGF secretion by TNBC cells and nerve fibre prolongation (Jin et al. 2023). When local electric shock occurs, e.g., during peritumoral electroacupuncture, both the density of sympathetic nerve fibres and the expression of NE are decreased, and the restoration of proapoptotic p75NTR expression impairs TNBC cell viability (Tian et al. 2022). Second, BC is sensitive to NGF, which activates multiple pathways. Typically, NGF increases VEGF secretion by BC cells, which in turn increases the nerve and vascular density in tumour lesions (Romon et al. 2010; Han et al. 2021). In addition, NGF promotes BC proliferation and migration by activating the Hippo pathway via the induction of the nuclear localization of YAP1, which can be blocked by phosphorylated large suppressor kinase 1 (LATS1) (Yang et al. 2019). A similar mechanism was also observed in cervical cancer (Wang et al. 2021a, b). NGF also mediates peritumoral neurite outgrowth by inactivating the RhoA and Rho-GTPase pathways, further aggravating the PNI of BC (Shang et al. 2012; Donato et al. 2023a, b). Third, the interaction between NGF receptors and adaptor proteins diversifies the role of NGF in cell fate determination. In TNBC, TrkA phosphorylates STAT3 at Y705, resulting in STAT3 nuclear transport and inducing the transcription of target genes, e.g., SOX2 and c-MYC (Regua et al. 2021). Binding to STAT3, CD44 isoforms, Ku70, and β1-integrin is necessary for maintaining cell proliferation, whereas the interactions between TNFR1-associated death domain protein (TRADD) and NF-κB have antiapoptotic effects on BC cells (Naderi et al. 2007; Trouvilliez et al. 2022; Nair et al. 2023; Com et al. 2007; Zhang et al. 2017; Donato et al. 2021).

Like in breast tissue, cyclical histological changes in the female reproductive system are also fine-tuned by fluctuating levels of female hormones. The menstrual cycle includes oocyte release and corpus luteum in the ovary, endometrial epithelial destruction and regeneration from the basalis layer (Massri et al. 2023). Under physiological conditions, NGF-mediated PG release is a necessary step for ovulation. During early follicular development, NGF promotes follicular cell proliferation and stimulates steroidogenesis (Streiter et al. 2016). Even though the neurotrophin pathway is inhibited during development from primordial to primary follicles, NGF induces the differentiation of primordial follicles into follicles, probably through interactions with granulosa and theca cells (Ernst et al. 2017; Retamales-Ortega et al. 2017). Abnormal expression of NGF has been observed in patients with benign gynaecological diseases. The serum NGF level is negatively correlated with the onset of pregnancy-induced hypertension (Lan et al. 2022). A low level of NGF in follicular fluid might indicate a diminished ovarian reserve, whereas increases in NGF levels and the nerve fibre density have been observed in patients with benign proliferative disease, e.g., at the lesion front of endometriotic lesions, suggesting an association between NGF and cell migration (Streiter et al. 2016; García-Solares et al. 2018). Epithelial ovarian cancer (EOC) accounts for approximately 80% of all ovarian cancer cases, with a 5-year relative survival rate of 31% for patients with advanced stages. Compared with the levels in normal ovaries, NGF and TrkA are significantly upregulated in EOC tissues, especially poorly differentiated EOC tissues (Campos et al. 2007; Garrido et al. 2022; Tapia et al. 2011). Like in BC, as EOC progresses, TrkA is upregulated, and p75NTR expression decreases gradually, probably because NGF-TrkA upregulates ADAM17, resulting in p75NTR shedding and degradation (Garrido et al. 2022). NGF increases the proliferation and angiogenesis of EOC by upregulating VEGF, and this regulatory axis might be maintained by follicle-stimulating hormone and mediated by NGF-induced expression of COX-2 and PG (Campos et al. 2007; Garrido et al. 2019, 2020a, b). This process could be disrupted by the application of metformin, probably by restraining the transcriptional function of c-MYC and β-catenin (Garrido et al. 2018, 2020a, b). Another molecular mechanism involves the upregulation of calreticulin (CRT) via the NGF-TrkA interaction. NGF also inhibits CRT translocation to the cell membrane to protect EOC cells from cytotoxic agent-induced endoplasmic reticulum stress (Vera et al. 2012, 2017).

### NGF and digestive system neoplasms

PDAC is the leading lethal malignancy of the digestive system, with a 5-year survival rate of less than 7% (Nomura et al. 2016). Because of the hidden position of the pancreas in the retroperitoneum, the diagnosis of PDAC remains challenging. Neuropathy is a common pathological feature of PDAC, e.g., an increased neural density and hypertrophy, pancreatic neuritis, and invasion of the intra- and extrapancreatic neural plexuses (Ceyhan et al. 2010). Consistent with this phenomenon, NGF is overexpressed in PDAC tissue compared with normal pancreatic tissue. A higher NGF level indicates a greater degree of pain, more frequent lymph node metastasis, and surgical margin involvement (Dang et al. 2006; Ma et al. 2008).

The mechanism of the interaction between PDAC cells and nerve cells has been studied via a coculture assay. In the binary coculture model, indirect communication between PDAC cells and nerve cells increased the proliferation, migration, and invasion of both types of cells. Another piece of evidence is that when nerve cells are cultured with conditioned medium isolated from PDAC cells, an increased neurite density is observed (Ceyhan et al. 2010; Liebl et al. 2013). In addition to the physicochemical properties, e.g., the glucose concentration in the supernatant, secretory proteins from PDAC parenchyma cells mediate the pathological generation of neurites via the external secretion of NGF (Li et al. 2015). Sonic hedgehog signalling molecule (SHH) from PDAC cells activates the hedgehog signalling pathway and increases NGF secretion and TrkA expression in nerve cells (Han et al. 2020). Exogenous HGF binds to the receptor c-MET, leading to the phosphorylation and nuclear localization of mTOR, which promotes NGF secretion from PDAC cells and the outgrowth of nerve cells (Qin et al. 2022; Nan et al. 2019). Endogenous NGF from PDAC cells in culture medium can be sustained by neurotransmitters, such as serine and NE, released from the sympathetic nervous system, exacerbating the activation of oncogenic pathways in PDAC cells, e.g., via ERK phosphorylation, AKT phosphorylation, and CD133 transport to the cytomembrane (Banh et al. 2020; Xin et al. 2016; Jiang et al. 2020a, b; Renz et al. 2018). When STAT3 is inactivated by the phosphorylation inhibitor AG490, the aforementioned neurotransmitter-mediated biological effect is abolished (Guo et al. 2013). In theory, NGF accumulates in cell supernatants via the symbiotic relationship of two cell types. Both PDAC and nerve cells express the NGF and TrkA proteins, likely resulting in positive feedback during the PNI process upon exposure to the initial pathogenic factors. In reality, the secretome of stroma cells complicates intercellular communication. Pancreatic stellate cells can release NGF, increasing the invasion and proliferation of PDAC cells (Lei et al. 2022). Another type of cell is Schwann cells (SCs), which dedifferentiate and proliferate upon axonal injury and secrete NGF proteins to expedite structural repair (Li et al. 2020). SCs can be activated by tumour-secreted proteins, e.g., TNFα, which is characterized by the upregulation of activation markers (e.g., c-Jun, glial fibrillary acidic protein, and p75NTR) and the cascade expression of NGF and TNFα (Salvo et al. 2021). SCs have a special affinity for PDAC cells, partly due to the paracrine effects of NGF and the concentration gradient (Demir et al. 2014). NGF contributes to autophagy in peritumoral SCs, and the latter are guided to PDAC cells, with enhanced myelin debris clearance and phagocytic abilities, aggravating the aberrant generation of axons and myelin (Li et al. 2020; Zhang et al. 2022a, b).

A similar mechanism could be observed in gastroenteric tumours. For example, NGF is overexpressed in gastric precancerous lesions, gastric cancer (GC), and CRC and maintains the proliferation of malignant cells (Lei et al. 2022; Zeng et al. 2021; Dou et al. 2018). The metastasis of CRC cells is promoted by the NGF-TrkA interaction and the subsequent phosphorylation of TrkA (Lei et al. 2022). CRC cells are also sensitive to HGF secreted by adipose stromal cells and release NGF proteins in response to the activation of STAT3 (Franco et al. 2021). SCs are also an important source of NGF that maintains the proliferation and metastasis of CRC cells by activating the MAPK/ERK cascade and EMT regulators, e.g., ZEB1 (Han et al. 2022). Neurotransmitters are oversecreted by nerve cells in CRC, and NE facilitates NGF secretion by CAFs and exacerbates intratumor sympathetic innervation (Kobayashi et al. 2024). Even though NE and its corresponding receptors, e.g., β2-adrenoceptors, are overactivated in gastroenteric tumour cells, parasympathetic nerves seem to predominantly promote cancer progression rather than sympathetic nerves (Sadighparvar et al. 2021; Qi et al. 2022). ACh is the key neurotransmitter and mediator that prompts malignant cells to secrete NGF. In this manner, NGF not only increases the recruitment of enteric nerves by stimulating axonogenesis but also enhances autocrine and paracrine processes to increase the progression of gastroenteric malignancies by activating the Hippos and Wnt pathways (Hayakawa et al. 2017; Monje 2017). Therefore, the exact NGF-related mechanisms depend on the different contexts. Whether NGF and its corresponding receptors are expressed could be one of the requirements for PNI. In cholangiocarcinoma (CCA) patients from Caucasian regions, NGF and TrkA are rarely detected in malignant epithelial cells, even though PNI is a classical route of metastasis for CCA, highlighting the potential role of other cell types in the microenvironment (Westphalen et al. 2019; Qian et al. 2024). The exact molecular mechanism of the PNI of NGF- and TrkA-negative tumours is worthy of further study.

### NGF and head-neck carcinoma

NGF is mainly secreted by the central and peripheral nervous system and immune cells, and most NGF is produced by submaxillary glands (Alzawi et al. 2022). Like in nerve tumours, NGF is upregulated in head-neck carcinoma, indicating a potential oncogenic role of NGF (Ye et al. 2011). HNSCC accounts for approximately 90% of the pathological type of head‒neck carcinoma and commonly develops in heterogeneous mucosal sites, e.g., the oral cavity, pharynx, larynx, and sinonasal cavity (Jiang et al. 2020a, b; Chung et al. 2018). The PI3K/AKT pathway is activated in response to NGF stimulation, which promotes the migration and perineural invasion of HNSCC cells (Alkhadar et al. 2020). Epidermal growth factor receptor (EGFR) is one of the key mediators of malignant behaviours and is upregulated in nearly 90% of HNSCCs, serving as a characteristic anticancer target (Morisse et al. 2023). The level of STAT3 phosphorylation is increased by NGF-TrkA signalling and exacerbates resistance to the EGFR inhibitor erlotinib (Lin et al. 2020). Another feature of HNSCC is that the effects of the overexpression and overaction of p75NTR by NGF are similar to those of TrkA, resulting in a more aggressive phenotype via the upregulation of the SLUG protein (Chung et al. 2018). The complex distribution of nerve fibres from the cranial nerve further reinforces the sensitivity of HNSCC lesions to NGF. HNSCC cells exhibit significant chemotaxis towards the trigeminal nerve and facial nerve. The communicating branch of two nerve trunks has been found at the sphenopalatine ganglion, the junction of the chorda tympani and the lingual nerve, and the parotid gland along the auriculotemporal branch of the mandibular nerve (Roh et al. 2015). Even worse, NGF secreted by HNSCC cells exacerbates local dysneuria, such as unbearable pain, logagnosia, and dysphagia (Ye et al. 2014). Theoretically, treatments targeting NGF would provide a maximal benefit for HNSCC patients because of their combined antiproliferative and analgesic effects. For oral cancer in animal models, anti-NGF mAbs impair the proper activation of NGF, decrease the volume of tumour lesions and relieve weight loss, accompanied by decreased levels of nociceptive receptors (Ye et al. 2011). When the selective COX-2 inhibitor celecoxib is combined with cetuximab, decreases in the level of NGF and inhibition of the MAPK/ERK pathway are observed, which provides a more accurate and effective strategy than monotherapy (Yang et al. 2015a, b). The differences in the expression of NGF with control tissue were summarized in Table 1.

**Table 1:** The differences in the expression of NGF with control tissue

Disease	NGF expression	Note
Acute ischaemic stroke	Upregulated	Detected in those with better outcomes
Alzheimer’s disease	Downregulated	Detected in CSF
Breast cancer	Upregulated	Detected in tissue
Colorectal cancer	Upregulated	Detected in tissue
Diminished ovarian reserve	Downregulated	Detected in follicular fluid
Endometriosis	Upregulated	Detected at the lesion front
Epithelial ovarian cancer	Upregulated	Detected in tissue
Gastric cancer	Upregulated	Detected in tissue
Gastric precancerous lesion	Upregulated	Detected in tissue
Glioma	Upregulated	Detected in tissue
Head and neck squamous cell carcinoma	Upregulated	Detected in tissue
Interstitial cystitis	Upregulated	Detected in urine
Major depressive disorder	Downregulated	Detected in tissue
Nocturnal enuresis	Upregulated	Detected in urine
Pancreatic ductal adenocarcinoma	Upregulated	Detected in tissue
Parkinson’s disease	Downregulated	Detected in CSF
Prostate cancer	Upregulated	Detected in tissue
Pseudorabies virus infection	Downregulated	Detected in tissue
Schizophrenia	Mostly Downregulated	Detected in tissue

*CSF* Cerebrospinal fluid

## NGF and cancer patient administration

### NGF and psychological management

Psychological problems usually occur in hospitalized patients, especially those with malignant disease, when they understand their serious condition. For those undergoing long-term antiproliferative treatment, depression might occur due to uncomfortable experiences and negative expectations (Xiong et al. 2022). The serum NGF level in outpatients with MDD is significantly lower than that in healthy controls (Maes et al. 2024). In addition, NGF might be associated with mild somatic symptoms, e.g., chronic fatigue syndrome and irritable bowel syndrome (Jonsjö et al. 2020; Chow et al. 2019). Extreme actions, e.g., suicide, are used to measure the severity of depression. Two studies involving autopsies revealed that NGF and TrkA levels were both decreased in the prefrontal cortex and hippocampal regions of the brain tissue of suicide victims (Dwivedi et al. 2005, 2009). However, the relationship between serum NGF levels and depression-related suicidality remains controversial, as Maes et al. supported this relationship, whereas two other independent studies raised objections Zhang et al. (2022); Dou et al. (2018); Di Franco (2021); Sadighparvar et al. (2021); Qi et al. (2022); Monje e al. (2017); Qian et al. (2024); Alzawi et al. (2022); Ye et al. (2011); Chung et al. (2018); Roh et al. (2015); Ye et al. (2014); Xiong et al. (2022); Maes et al. (2024); Jonsjö et al. (2020); Chow et al. (2019); Dwivedi et al. (2005, 2009); Bilgiç et al. (2020); Maes et al. (2023); Wiener et al. (2015). In addition to the different age ranges of the included patients, the reason for this difference might be the NGF expression pattern, as a decrease in NGF levels might have occurred within a short period before the decision to commit suicide, which could be partly attributed to NGF downregulation in the brains of the decedents. Despite the lack of a sufficient theoretical basis, during hospitalizations, decreased serum or plasma NGF levels should be monitored because of the potential risk for dangerous events (e.g., suicide, violence, and neuroticism) and the need for a psychological crisis intervention. However, some exceptions could not be explained. First, NGF could be upregulated in paediatric patients, e.g., children with autism spectrum disorder, probably because of the positive correlation between NGF and the overall developmental status of children. Additionally, no differences were found between children with low and high suicidality. The diagnostic value of decreased NGF levels might be applicable only to adults (Bilgiç et al. 2020; Liu et al. 2021a, b; Gevezova et al. 2022; Karayağmurlu et al. 2019). Second, when dysphrenia further intensifies, e.g., the conversion from MDD to bipolar disorder, NGF levels often increase, which might be confusing if no specialized medical history or auxiliary examination is provided (Pedrotti Moreira et al. 2019). Third, if a patient suffers from mental disorders as well as NGF-secreting neuroendocrine neoplasms, determining the trend for the variation in NGF levels is difficult (Zhang et al. 2022a, b). Additional related clinical and translational studies are needed.

### NGF and antinociceptive treatment

Considering that the NGF-TrkA interaction is involved in pain modulation, two strategies, i.e., targeting NGF or TrkA, have been developed. Selective and nonspecific allosteric TrkA inhibitors have been designed and shown to inhibit NGF-induced neurite outgrowth and regulate arthrodynia (Subramanian et al. 2021; Bagal et al. 2018). Anti-NGF mAbs, including tanezumab, fulranumab, and other antibodies with only preclinical data, e.g., DS002, huAb45, and 58F10G10H, which function by sequestering NGF from TrkA in a dose-dependent manner, have been widely studied (Liang et al. 2022; Liu et al. 2022a, b; Bannwarth and Kostine 2017; Dong et al. 2022). In cancerous lesions, certain types of tumour cells secrete NGF, which leads to sensory and sympathetic nerve pathological sprouting and the formation of a neuroma-like structure (Mantyh et al. 2010). For some patients with advanced tumours, tumour-derived NGF causes bone tissue remodelling, aggravating local bone pain and pathological fracture (Jing et al. 2023). Therefore, anti-NGF mAbs should be applied pre-emptively before histological changes in nerves and bones occur, but compensatory administration could also attenuate cancer pain (Jimenez-Andrade et al. 2011). In theory, the antinociceptive effects of anti-NGF mAbs could be better than those of transitional nonsteroidal anti-inflammatory drugs (NSAIDs), but few clinical trials have reported any advantages. Sopata et al. reported that long-term treatment with tanezumab as a complement to daily opioid use slightly relieved metastatic bone cancer pain (10 mg for 8 weeks, up to 40 weeks) (Sopata et al. 2015). Slatkin et al. studied fulranumab (9 mg for 4 weeks), yet no significant improvement in antinociception was observed (Slatkin et al. 2019). Actually, early in 2010, despite acceptable tolerance and a low discontinuation rate (less than 5–10%), clinical trials of anti-NGF mAbs were stopped by the US Food and Drug Administration (FDA) due to serious adverse effects, including neurological problems (e.g., paraesthesia, hypoesthesia, and extremity pain) and joint-related problems (e.g., arthralgia in the knee and rapidly progressive osteoarthritis) (Bannwarth and Kostine 2017). A possible reason might be the comprehensive deprivation of the NGF downstream pathway, especially its neurotrophic and neuroprotective functions. Several technical issues, such as improving antibody specificity, maintaining structural stability in tissue, and avoiding nonfunctional binding to NGF, should be addressed (Porte et al. 2014). Additionally, the application of anti-NGF mAbs is restricted by social economics. At the present stage, commercialized monoclonal antibodies require expensive production lines and complex approval processes. Even worse, compared with NSAIDs, anti-NGF mAbs might be overdosed due to uncontrollable biodegradation caused by macromolecular immunogenicity, eventually increasing the cost of therapy.

Nonetheless, anti-NGF mAbs are worthy of further investigation for the following reasons. First, NGF maintains sufficient expression of µ-opioid receptors in the central nervous system. Hence, the intrathecal delivery of NGF enhances the analgesic effect of fentanyl on diabetes-related hyperalgesia, which could explain why anti-NGF mAbs exhibit limited antinociceptive effects (Shaqura et al. 2013). The maintenance of pain receptors or their regulators by other methods should be considered when anti-NGF mAbs are designed, such as by coupled drugs and epitope replacement. Second, strategies targeting NGF probably have additional antineoplastic effects to improve the patient prognosis. NGF blockade impairs the proliferation of oral cancer cells and relieves the general condition of murine oral cancer models (Dou et al. 2018). In addition to its effects on bone sarcoma, NGF blockade also significantly inhibits bone destruction and skin hypersensitivity in animal models (McCaffrey et al. 2014; Guedon et al. 2016; Hou et al. 2024). Targeting NGF is also a potential supplement to traditional anticancer treatment. Anti-NGF mAbs alleviate the peripheral neuropathy caused by chemotherapeutic drugs such as paclitaxel, cisplatin and vincristine (Da Vitoria Lobo et al. 2024; Liang et al. 2022). Upon doxorubicin chemotherapy, the reactive upregulation of NGF exacerbates drug resistance and cancer pain, highlighting the importance and urgency of targeting NGF (Zuo et al. 2024). Third, the precise application of NGF antibodies and inhibitors has been poorly studied. The high affinity for NGF and antinociceptive effects of these agents must be independent of their neurotrophic effects to prevent serious systemic adverse reactions due to nerve damage. One of the important reasons is the insufficient understanding of the pathogenic molecular mechanism of each subgroup of patients, especially the unconventional downstream pathways. For example, cross-sensitization mediated by other proteins, e.g., endothelin-1, should be investigated in patients with sustained cutaneous pain (Khodorova et al. 2018).

### NGF and wound healing

For the patients with malignancies, tissue injuries always occur, such as primary injury by the invasion of cancerous lesions, and secondary injuries by invasive therapeutic manipulation (e.g., skin incision and radical surgery). Tissue damage is followed by a timely wound healing process, including epithelization, fibrogenesis, innervation, and vascularization with unknown precedence orders, unless negative factors, e.g., malnutrition, infection, and nonspecific inflammation, exist (Giuliani et al. 2020). When injury to the body surface or accessible deep tissue occurs, wound healing can be expedited by debridement, suturing, and drug intervention. Local NGF administration is an excellent example, especially when nerve injury occurs simultaneously. Unlike in cancerous lesions, NGF-associated pathways are protective when injuries occur. The transdermal application of NGF variants accelerates the recovery of diabetic ulcers by activating the AKT pathway with few hyperalgesic effects (Giuliani et al. 2020). Similarly, the transdermal injection of NGF into endochondral regions and tibial fracture calluses activates the Wnt pathway and integrin pathway, contributing to increased trabecular number, connective density, and bone mineral density and ultimately increasing bone healing (Rivera et al. 2020, 2023). Wnt ligands, e.g., Wnt5a, promote the proliferation of SCs and the secretion of NGF and VEGF (Liu et al. 2022a, b). In some studies, nerve regeneration has been shown to be a leading factor contributing to wound healing compared with epithelial and capillary regeneration. The healing of nerve fibres can be measured by axonal growth, myelination, electrophysiological recovery, target organ innervation, and motor function recovery (Liu et al. 2022a, b). For example, erectile dysfunction is a common problem for cryotherapy and radical prostatectomy of PC due to concomitant injury of the cavernous nerve. This injury can be relieved by the implantation of adipose-derived stem cells and bioactive fibrous membranes. The former increases the production of NGF to inhibit apoptotic mediators, e.g., cleavage of Caspase 3, whereas the latter selectively binds and captures uNGF (Yang et al. 2015a, b; Casanova et al. 2023). Similarly, transplanting transection-activated sural nerve fascicles into the brains of PD patients could result in neuroprotective effects via NGF secretion and apoptosis inhibition (Chau et al. 2022). In this sense, local NGF enhances the adaptive response, as manifested by the upregulation of TrkA on the surface of nerve cells (Cho et al. 2020).

The poor functional status of local nerve cells may be attributed to multiple factors. During development, NGF expression is promoted in a nerve-independent manner, whereas NGF levels and nerve density decrease synchronously with age (Mahdee et al. 2019). Chronic inflammation delays wound healing caused by TNF-α induced by infectious or ischaemic factors. In skin wounds, keratinocytes secrete NGF spontaneously. TNF-α further upregulates NGF expression and increases the distribution of NE receptors, which are sensitive to proinflammatory sympathetic signals and unduly increase NGF production in response, aggravating chronic injuries and pain (Wijaya et al. 2020a, b). Antiproliferative treatments, such as radiation for nasopharyngeal carcinoma and doxorubicin for breast cancer, also have side effects such as central neurotoxicity and cognitive dysfunction, which can be alleviated by the active and passive upregulation of NGF, e.g., the NGF plus steroids regimen and Carissa macrocarpa extracts, respectively (Liu et al. 2020; Orabi et al. 2021). Nevertheless, most conclusions are based on animal studies and in vitro assays. More clinical trials are needed.

## Advances in NGF applications

The diagnostic value of NGF has been studied. In addition to being detected in the peripheral blood and CSF, NGF can be detected in the saliva. Salivary NGF is downregulated in adults with temporomandibular disorder and some obese children (Jasim et al. 2020; Tvarijonaviciute et al. 2020). A neural cell-cell interaction microchip (NCCIM) was developed to measure NGF levels in real time upon coculture between nerve cells and other target cells, providing convenience for secretome mechanistic research (Abdullah et al. 2020).

Traditional surgical and drug delivery approaches, e.g., transdermal patches, biological membranes, surgical staplers, and absorbable sutures, might be options for loading NGF to address the anastomotic stoma after radical resection of malignancies, traumatic organ damage, and skin ulceration induced by internal diseases or chemoradiotherapy. NGF can be immobilized and purified from mixtures via chromatography or MG-PEG-NTA-Ni + 2 nanocomposites (Khataminezhad et al. 2023; Gu et al. 2020). However, natural NGF fails to exhibit optimal biological effects and drug distributions, inspiring an attempt to modify the NGF structure. For example, the NGF derivative R100E could facilitate the healing of nerve endings and skin lesions in individuals with diabetes (Giuliani et al. 2020). The NGF structure has also been simplified, e.g., the acetylated human NGF 1–14 sequence (hNGF1-14), to overcome drug delivery restrictions mediated by the BBB (Triaca et al. 2020). New delivery by artificial synthetic materials, e.g., liposomes incorporating cardiolipin and phosphatidic acid, contributes to NGF transport through the BBB (Kuo et al. 2019). Poly (ethylene glycol) dimethacrylate microparticles loaded with NGF have been developed to treat fractures (Rivera et al. 2023). Specific siRNAs targeting NGF are widely studied in PDAC. Gold nanocluster- and cationic perfluorocarbon nanoemulsion-assisted delivery to PDAC lesions overcomes the disadvantages of siRNAs, such as a short circulation lifetime, low tissue specificity, poor tumour penetration, and poor cellular uptake (Lei et al. 2017; Ding et al. 2022). Bioactive membranes can be implanted into orthotopic lesions or injuries, including polycaprolactone membranes, six bilayers of heparin/collagen membranes, and electroresponsive biohybrid membranes made from Bombyx mori silkworm fibroin and reduced graphene oxide, all of which are able to adsorb and carry NGF, facilitating SC adhesion and neurogenesis (Casanova et al. 2023; Magaz et al. 2020; Pinzon-Herrera et al. 2020).

Gene therapy targeting NGF is also an option for local treatment, as recombinant mouse NGF has limited biological activity compared with that of human NGF. Human NGF gene vectors are transfected into murine submandibular glands to establish a human NGF-producing model (Gu et al. 2020). Three clinical trials have been conducted on NGF gene therapy for AD. Tuszynski et al. implanted modified autologous fibroblasts, whereas Eriksdotter-Jönhagen et al. implanted an NGF biodelivery device, NsG0202, into the forebrains of AD patients, and the degree of cognitive decline was significantly improved (Tuszynski et al. 2005; Eriksdotter-Jönhagen et al. 2012). In contrast, Rafii et al. transfected an adeno-associated virus vector carrying NGF into the nucleus basalis of Meynert via intracerebral injection, but no improvement was observed, suggesting that potential nonspecific transfection targets and puncture sites should be adjusted (Rafii et al. 2018). Even though the product could be synthesized and released in a limited area, this technique remains to be improved for better safety and more accurate targeting. The clinical trials about NGF and TrkA were summarized in Table 2 (Zhang et al. 2025; Pang et al. 2023; Munkholm and Arendt-Nielsen 2017; Castle et al. 2020; Falsini et al. 2016; Kim et al. 2015; Rafii et al. 2014; Martino et al. 2013; Blandini et al. 2006; Lambiase et al. 2007; Roblin et al. 2015; Watt et al. 2019).

**Table 2:** The clinical trials about NGF and TrkA

Authors	Country	Year	Type	Disease	Study object	Topic	Conclusion	References
Zhang et al.	China	2025	Prospective	Healthy participant	NGF	Anti-NGF monoclonal antibody	The safety and tolerability of the antibody AK115 was favorable	Zhang et al. (2025)
Pang et al.	China	2023	Retrospective	Interstitial cystitis	NGF, TrkA	Biomarker	NGF and TrkA were both independent prognostic factors	Pang et al. (2023)
Castle et al.	USA	2020	Retrospective	Alzheimer’s disease	NGF	NGF delivery	Failed AAV delivery to specific encephalic region might be the cause of death	Munkholm (2017)
Slatkin et al.	USA	2019	Prospective	Cancer	NGF	Anti-NGF monoclonal antibody	Fulranumab failed to improve the antinociception effect	Slatkin et al. (2019)
Watt et al.	United Kingdom	2019	Prospective	Knee osteoarthritis	TrkA	TrkA inhibitor	TrkA inhibitor ASP7962 failed to improve pain or physical function of knee joint	Watt et al. (2019)
Rafii et al.	USA	2018	Prospective	Alzheimer’s disease	NGF	NGF delivery	No improvement was observed by transfecting AAV vector carrying NGF	Rafii et al. (2018)
Falsini et al.	Italy	2016	Prospective	Retinitis pigmentosa	NGF	NGF preparation	The safety and possible efficacy of NGF eye-drops was favourable	Falsini et al. (2016)
Munkholm et al.	Denmark	2016	Prospective	Pain	NGF	Antinociceptive effect	Acidic stimulation aggravates NGF-induced hyperalgesia in muscle tissue	Castle et al. (2020)
Kim et al.	Korea	2015	Prospective	Overactive bladder syndrome	NGF	Biomarker	Urinary NGF is potential biomarkers for therapeutic markers	Kim et al. (2015)
Roblin et al.	United Kingdom	2015	Prospective	Psoriasis	TrkA	TrkA inhibitor	TrkA inhibitor CT327 relieved the pruritus.	Roblin et al. (2015)
Sopata et al.	Poland and USA	2015	Prospective	Metastatic bone cancer	NGF	Anti-NGF monoclonal antibody	Tanezumab slightly relieved metastatic bone cancer pain	Sopata et al. (2015)
Rafii et al.	USA	2014	Prospective	Alzheimer’s disease	NGF	NGF delivery	NGF gene therapy is well-tolerated and feasible by bilateral stereotactic administration	Rafii et al. (2014)
Martino et al.	Italy	2013	Prospective	Major depressive disorder	NGF	Biomarker	Serum NGF is potential biomarkers for therapeutic markers	Martino et al. (2013)
Eriksdotter-Jönhagen et al.	Sweden	2012	Prospective	Alzheimer’s disease	NGF	NGF delivery	Cognitive decline was relieved bt implanting NGF biodelivery devices	Eriksdotter-Jönhagen et al. (2012)
Lambiase et al.	Italy	2007	Prospective	Neurotrophic keratopathy	NGF	NGF preparation	NGF eye-drops contribute to ulcer healing and no circulating NGF antibodies was found	Lambiase et al. (2007)
Blandini et al.	Italy	2006	Observational	Headache	NGF	Biomarker	NGF was potentially involved in the pathophysiology of primary headaches	Blandini et al. (2006)
Tuszynski et al.	USA	2005	Prospective	Alzheimer’s disease	NGF	NGF delivery	Cognitive decline was relieved by implanting modified autologous fibroblasts	Tuszynski et al. (2005)

*AAV* Adeno-associated virus vector

## Conclusions and future perspectives

**Fig. 4:**
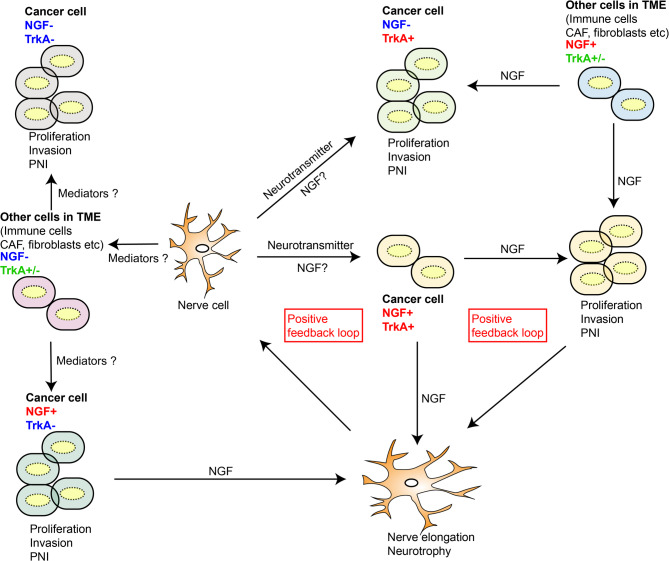
Hypothesis of the potential molecular mechanisms according to the expression level of NGF and TrkA receptor

NGF serves as a tumour-promoting factor, PNI mediator and indicator of a poor prognosis for patients with cancerous lesions. The biological effect of NGF depends on the sensitivity of target cells, which can be evaluated by two factors, i.e., NGF levels in the microenvironment and the NGF receptor distribution in target cells (Fig. 4). The sources of NGF include NGF-secreting tumour cells, peritumoral stromal cells, immune cells, and hyperfunctional nerve cells, leading to a primary increase in local NGF levels. When tissue injuries occur, stress responses induce the release of several cytokines and mediators and subsequently stimulate secondary NGF secretion from the SCs to prolong damaged nerve fibres (Tazawa et al. 2021). Compared with primary upregulation, secondary upregulation may be accompanied by more severe nerve injuries, significant neural symptoms, and positive detection of damage biomarkers, e.g., tau protein and chitotriosidase in CSF (Cheung et al. 2018). However, whether the invasion of nerve fibres results in the compensatory expression of NGF is still unknown.

**Fig. 5:**
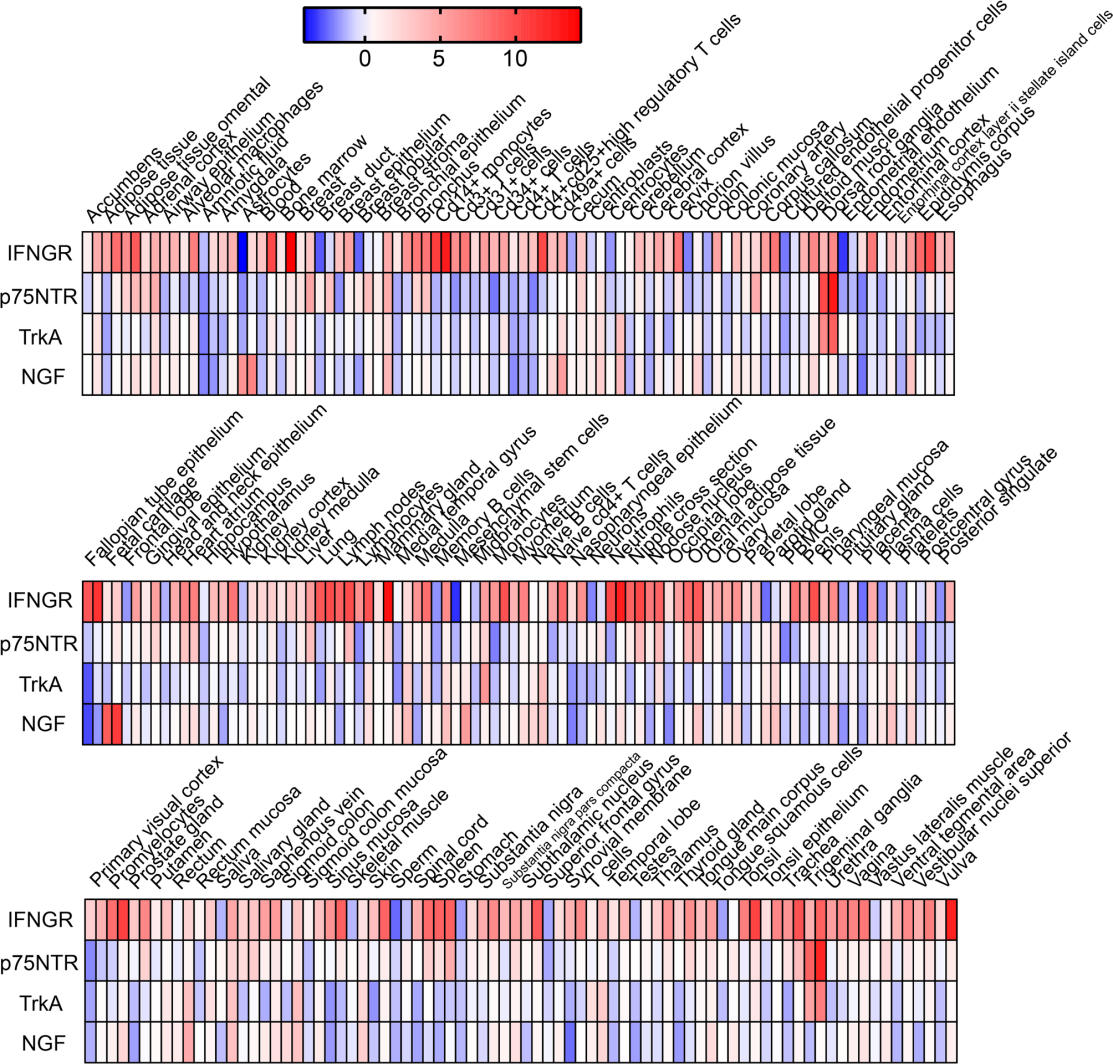
Presentation of the expression level of NGF and NGF receptors in different tissue. IFNGR is widely expressed in human tissues. Hence, IFNGR is selected for positive control. The expression profiles were contributed by McCall et al. and downloaded from BioGPS database (http://biogps.org/). The gradual change from red to blue represents changes in gene expression from high to low

The membrane abundance of TrkA can be regulated flexibly by ProNGF and NGF, but the main determining factor is the tissue type. NGF and TrkA abundance should be measured before NGF-related antiproliferative therapy is administered (Fig. 5) (McCall et al. 2011; Wu et al. 2016). For malignancies characterized by the positive expression of NGF and TrkA, the communication between nerve cells and cancer cells forms a positive feedback loop by conventional autocrine and paracrine signalling, leading to the rapid progression of PNI and distal metastasis. In contrast, the progression of malignancies with negative expression of NGF and positive expression of TrkA can also be promoted, but the feedback mechanism is ambiguous. In theory, malignancies with little expression of NGF and TrkA can be regulated by secreted proteins but not neurotrophins. This extracellular protein might be released from either nerve cells or other downstream cells that communicate with nerve cells.

NGF has functions in both promoting tumour progression and maintaining the homeostasis of normal structures. Hence, the systematic application of NGF or anti-NGF for any purpose may not be the optimal solution, even though murine NGF has been allowed for preventing optic nerve injury and N-hexane poisoning via intramuscular injection. First, systematic application increases the probability of neutralizing autoantibody formation, impairing the therapeutic effect to some degree. Second, TrkA is widely expressed, such that any change in the overall NGF level is a possible cause of adverse events, e.g., fever, pruritus, erythema, muscular pain, diarrhoea, and liver dysfunction. Third, NGF application might be a potential cause of malignant transformation, albeit with no direct evidence available at present. Local treatment should be focused on because of its advantages of timely, specific, and adaptive effects on the physiological NGF function rhythm. However, the detailed mechanisms of the TrkA-dependent and -independent effects of NGF are not fully understood, especially the key factors that determine the direction of flow to either oncogenic or neurotrophic pathways, the exact role of perifocal participants in cellular communication, and the reason for the differences in NGF expression among different organs. More clinical trials and development projects are welcomed for better application of NGF, a pleuripotent but conserved cytokine.

## Data Availability

The data used in this review can be downloaded from BioGPS database (http://biogps.org/).

## Abbreviations

ACh: Acetylcholine
AD: Alzheimer’ s disease
ADT: Androgen deprivation therapy
Anti-NGF mAbs: NGF neutralizing antibodies
APP: Amyloid precursor protein
AR: Androgen receptor
ASIC3: Acid-sensing ion channel 3
Aβ: β-amyloid
BBB: Blood-brain barrier
BDNF: Brain-derived neurotrophic factor
Bmf: Bcl-2 modifying factor
CAF: Cancer-associated fibroblast
CAMK2B: Calcium/calmodulin-dependent protein kinase II beta
CCA: Cholangiocarcinoma
CHRM4: Cholinergic receptor muscarinic 4
CNS: Central nervous system
COX-2: Cyclooxygenase-2
CRC: Colorectal cancer
CRD: Cysteine-rich domain
CREB: cAMP/cAMP responsive element binding protein
CRT: Calreticulin
CSF: Cerebrospinal fluid
DHEA: Dehydroepiandrosterone
DRG: Dorsal root ganglion
ECD: Extracellular domain
EGFR: Epidermal growth factor receptor
EMT: Epithelial-mesenchymal transition
EOC: Epithelial ovarian cancer
ER: Estrogen receptor
ERK1/2: Extracellular signal-regulated kinase 1/2
FDA: The US food and drug administration
GBM: Glioblastoma multiforme
GC: Gastric cancer
GnRH: Gonadotropin-releasing hormone
HER2: Human epidermal growth factor receptor 2
HGF: Hepatocyte growth factor
hnRNP: Heterogeneous nuclear ribonucleoprotein
HNSCC: Head and neck squamous cell carcinoma
HSANs: Hereditary sensory and autonomic neuropathies
ICD: Intracellular domain
IP_3_: Inositol triphosphate
JAK: Janus kinase
LATS1: Large suppressor kinase 1
MAPK: Mitogen-activated protein kinase
MDD: Major depressive disorder
MMP: Matrix metalloproteinase
mTOR: Mechanistic target of rapamycin kinase
NB: Neuroblastoma
NCCIM: Neural cell-cell interaction microchip
NE: Norepinephrine
NEPC: Neuroendocrine differentiation
NGF: Nerve growth factor
NSAID: Nonsteroidal anti-inflammatory drug
NSCLC: Non-small cell lung cancer
NT-3: Neurotrophin-3
p75NTR: p75 neurotrophin R
PC: Prostate cancer
PDAC: Pancreatic ductal adenocarcinoma
PG: Prostaglandin
PI3K: Phosphoinositide-3 Kinase
PLC-γ: Phospholipase C-γ
PNI: Perineural invasion
PR: Progesterone receptor
ROS: Reactive oxygen species
SC: Schwann cell
SHH: Sonic Hedgehog signalling molecule
STAT: Signal transducer and activator of transcription
TEAD1: TEA domain family member 1
TLR: Toll-like receptor
TM: Transmembrane domain
TME: Tumour microenvironment
TNBC: Triple-negative breast cancer
TNF: Tumour necrosis factor
TRADD: TNFR1-associated death domain protein
TRAF4: TNF receptor associated factor 4
TRAIL: Tumour necrosis factor-related apoptosis-inducing ligand
TrkA: Neurotrophic tropomyosin receptor kinase A
TRPV1: Transient receptor potential cation channel 1
VCAM-1: Vascular cell adhesion molecule-1
VEGF: Vascular endothelial growth factor
YAP1: Yes-associated protein 1
ZBTB46: Zinc finger and BTB domain containing 46
ZEB1: Zinc finger E-box binding homeobox 1
